# Pathogenesis-directed therapy of methylphenidate-induced oxidative heart damage in rats

**DOI:** 10.3389/fphar.2024.1503032

**Published:** 2025-01-03

**Authors:** Izzet Emir, Seval Bulut, Bahadır Suleyman, Renad Mammadov, Nurinisa Yucel, Betul Cicek, Gulce Naz Yazici, Durdu Altuner, Murat Gunay, Halis Suleyman

**Affiliations:** ^1^ Department of Cardiovascular Surgery, Faculty of Medicine, Erzincan Binali Yıldırım University, Erzincan, Türkiye; ^2^ Department of Pharmacology, Faculty of Medicine, Erzincan Binali Yildirim University, Erzincan, Türkiye; ^3^ Pharmacy Services Program, Vocational School of Health Services, Erzincan Binali Yildirim University, Erzincan, Türkiye; ^4^ Department of Physiology, Faculty of Medicine, Erzincan Binali Yildirim University, Erzincan, Türkiye; ^5^ Department of Histology and Embryology, Faculty of Medicine, Erzincan Binali Yıldırım University, Erzincan, Türkiye; ^6^ Biochemistry Laboratory, Erzincan Mengücek Gazi Training and Research Hospital, Erzincan Binali Yildirim University, Erzincan, Türkiye

**Keywords:** adenosine triphosphate, melatonin, methylphenidate, metyrosine, oxidative stress

## Abstract

**Aim:**

The current study aimed to investigate the protective effects of adenosine triphosphate (ATP), metyrosine, and melatonin on possible methylphenidate cardiotoxicity in rats using biochemical and histopathological methods.

**Methods:**

Thirty rats were separated into five groups: healthy (HG), methylphenidate (MP), ATP + methylphenidate (ATMP), metyrosine + methylphenidate (MSMP), and melatonin + methylphenidate (MLMP). ATP (5 mg/kg) was given intraperitoneally once daily, metyrosine (50 mg/kg) orally twice daily, and melatonin (10 mg/kg) orally once daily. Methylphenidate (10 mg/kg) was administered orally once daily for 1 h after ATP, metyrosine and melatonin. The protocol was repeated for 30 days. Subsequently, blood samples were taken from the tail veins of the animals to measure adrenaline, noradrenaline, dopamine, troponin I (TP I) and creatine kinase MB (CK-MB) levels; the animals were then euthanized and the heart tissues were extracted. Tissues were analyzed for malondialdehyde (MDA), total glutathione (tGSH), superoxide dismutase (SOD), and catalase (CAT) and histopathologically.

**Results:**

In MP group, MDA, adrenaline, noradrenaline, dopamine, TP I, and CK-MB levels increased (*p* < 0.001) and tGSH, SOD, and CAT levels decreased (*p* < 0.001) compared to HG, and histopathologic damage developed. Oxidant levels were lower and antioxidant levels were higher in ATMP, MSMP, and MLMP groups compared to MP group (*p* < 0.001). Catecholamine levels were measured lower in the MSMP group compared to the MP group (*p* < 0.001). TP I and CK-MB levels were lower in ATMP, MSMP and MLMP groups compared to MP (*p* < 0.05), with the lowest being in rats given ATP (*p* < 0.001). ATP, melatonin, and metirozin applications were effective to different degrees in preventing histopathological changes.

**Conclusion:**

This study may guide clinical trials using ATP and melatonin to prevent methylphenidate-induced myocardial injury.

## 1 Introduction

Methylphenidate is a piperidine derivative central nervous system stimulant, which has a structural similarity to amphetamine (Trenque et al., 2014). It is a psycho-stimulant used in the 1st-line therapy of “Attention Deficit Hyperactivity Disorder” (ADHD) in children and adults and the 2nd-line therapy of “Narcolepsy” in adults (Bieś et al., 2023). Methylphenidate treatment for children and adults with suspected attention deficit disorder has been reported to increase cognitive skills and improve psychological functioning and performance (Senior et al., 2023). Methylphenidate produces this biological effect by inhibiting the reuptake of norepinephrine and dopamine through inhibition of the dopamine and norepinephrine transporter in presynaptic neurons (Bieś et al., 2023). It has also been shown that alpha-1 adrenergic receptor activity mediates methylphenidate’s beneficial effects on maintaining attention (Tilley and Gu, 2008). Another direct effect of methylphenidate that contributes to increased extracellular dopamine and norepinephrine concentrations is its agonist role at the serotonin 1A receptor (5-HT1A) (Markowitz et al., 2009).

Although most studies have demonstrated that methylphenidate is a well-tolerated psychostimulant, side effects of varying severity may develop depending on the dosage and level of drug abuse. The most common side effects associated with methylphenidate are headaches, insomnia, nervousness, palpitations, tachycardia, nasal congestion, nausea, cough, and skin rashes (Bieś et al., 2023; Senior et al., 2023; Trenque et al., 2014). Cardiotoxicity is an uncommon but potentially serious side effect (Liang et al., 2018). An increased cardiac workload, chronic excessive sympathetic activity, hypertension, and endothelial dysfunction may predispose to left ventricular hypertrophy and arrhythmia attacks (Lamberti et al., 2015; Torres-Acosta et al., 2020). Concern about the cardiovascular safety of methylphenidate and other psychostimulant agents persists, as conflicting findings have been reported in studies over the years (Hennissen et al., 2017). Schelleman et al. (2012), however, failed to establish a causal relationship between methylphenidate and the risk of serious cardiovascular events (Schelleman et al., 2012). In later studies, the cardiotoxic effects of amphetamine-like substances have been associated with oxidative stress, cardiomyocyte necrosis, abnormal cardiac protein synthesis, and abnormal intracellular calcium levels (Jafari Giv, 2017). Moreover, long-term use of methylphenidate has been linked to low ejection fractions and heart failure (Ugwendum et al., 2024). Cardiomyopathy as one of the main causes of heart failure in the literature (Liu et al., 2016; Reddy et al., 2020). Various factors such as genetic factors and viral infections have been held responsible for the pathogenesis of cardiomyopathy. In addition, Liu et al. drew attention to intracellular energy levels in myocytes for cardiomyopathy and stated that imbalances would disrupt the biological activities of myocytes emphasized (Liu et al., 2016).

It is well known that cardiomyocytes require a large amount of energy in order to maintain their normal biological functions. It has been reported that intracellular adenosine triphosphate (ATP) protects myocyte function and that potassium-dependent decreases in ATP levels may lead to cardiomyopathy. The role of ATP in maintaining the normal function of cardiac tissues is crucial (Liu et al., 2016). ATP regulates its biological activity through purinergic receptors. The role of purinergic signaling in cardiovascular diseases has also been mentioned in the literature (Sudi et al., 2023). On the other hand, the roles of ATP include its involvement in the synthesis of antioxidants that remove reactive oxygen species (ROS) and its use as an energy source during synthesis (Saquet et al., 2000; Yi et al., 2010). In the literature, it has been pointed out that methylphenidate impairs the activity of enzymes in the electron transport chain and decreases ATP levels (Foschiera et al., 2022). On the other hand, overstimulation of the sympathetic nervous system has been held responsible for the pathogenesis of methylphenidate cardiotoxicity (Torres-Acosta et al., 2020). In addition to increasing blood pressure and heart rate, the direct effects of catecholamine increase on cardiac myocytes may lead to cardiotoxicity. It has been shown that myocardial overexpression of norepinephrine-induced beta-1 receptors may lead to myocyte apoptosis and cardiomyopathy. Furthermore, stimulation of alpha-1 receptors has also been associated with myocyte hypertrophy. Therefore, it is likely that catecholamines may lead to pathological remodeling and impairment of contractile function (Dadfarmay and Dixon, 2009). Methyrosine is a tyrosine hydroxylase inhibitor and therefore limits catecholamine synthesis. In addition to its sympatholytic effects, the antioxidant properties of metyrosine were the reason for the inclusion of metyrosine in this study (Ahiskalioglu et al., 2015). To obtain more comprehensive and robust evidence for the pathogenesis of the possible cardiotoxic effect of methylphenidate, melatonin was also used in the study. Melatonin (N-acetyl-5-methoxytryptamine) is the major hormone of the pineal gland. It regulates the hypothalamic-pituitary axis through the hypothalamus (Ahmed et al., 2005). The ability of melatonin to reduce oxidative stress, pyroptosis, and apoptosis through upregulation of the Sirt1/Nrf2 pathway has been previously noted (Zhang et al., 2023). In addition, the ability of melatonin to reduce catecholamine synthesis directly and indirectly via cAMP has been highlighted (Komatsubara et al., 2017). For these reasons, the present investigation was designed to see the effects of methylphenidate on the hearts of rats and to evaluate the protective effects of ATP, metirosine, and melatonin in case of possible damage. It was also intended to evaluate the role of oxidants, antioxidants, adrenaline, noradrenaline, and dopamine in the pathogenesis of possible methylphenidate-induced cardiotoxicity.

## 2 Material and method

### 2.1 Animals

Thirty albino Wistar rats (265–280 g) were used in this investigation and were obtained from the Experimental Animal Research and Application Centre of Erzincan Binali Yildirim University. One week before the experiment, rats were brought into the laboratory for acclimatization and housed in groups of six rats in rooms with a room temperature of 22°C and a 12-h lighting cycle. Animals were fed normal pellet feed and tap water *ad libitum* during this period.

### 2.2 Chemical substances

The thiopental sodium was produced by, IE Ulagay (Türkiye), ATP was by Zdorove Narodu (Ukraine), metyrosine Sigma Chemical (Munich, Germany), melatonin by Przedsiebiorstwo Farmaceutczne LekAm (Poland), and methylphenidate (JOHNSON and JOHNSON) by Istanbul, Türkiye.

### 2.3 Experimental groups

The five groups of six rats are as follows: healthy (HG), methylphenidate (MP), ATP + methylphenidate (ATMP), metyrosine + methylphenidate (MSMP), and melatonin + methylphenidate (MLMP).

### 2.4 Experimental procedures

Drug solutions were prepared separately for each treatment session throughout the experiment. Tablets containing 18 mg methylphenidate were dissolved in pure water at 6 cc per tablet. A solution was prepared containing 3 mg methylphenidate in 1 cc (1 cc for 10 mg/kg per rat). 110 mg of metyrosine in powder form was dissolved in 8 cc of distilled water and a solution containing 13.75 mg per 1 cc was prepared (1 cc for 50 mg/kg per rat). To prepare melatonin solution, 4 tablets containing 5 mg of melatonin each (20 mg) were dissolved in 7.5 cc distilled water. A solution containing 2.7 mg melatonin in 1 cc was prepared (1 cc for 10 mg/kg per rat). In experimental animals, differences in body surface area and metabolism may cause drug doses to differ from those in humans. Various methods can be used to convert animal doses to human doses (Nair and Jacob, 2016). However, the doses in this study were determined by reference to previous studies.

In the ATMP group, ATP 4 mg/kg was given intraperitoneally (ip) once daily (Dagel et al., 2024). The MSMP group received metyrosine (50 mg/kg) orally twice daily by gavage (Albayrak et al., 2011). In the MLMP group, gavage administered melatonin (10 mg/kg) orally once daily (Amer et al., 2021). The same amount of distilled water was given orally to the HG and MP groups. All rat groups except HG were administered 10 mg/kg methylphenidate orally once a day 1 h following the administration of ATP, metyrosine, melatonin, and distilled water (Iqbal et al., 2019). For 30 days, this procedure was repeated. After this period, 2.5 cc blood samples were taken from the animals’ tail veins without anesthesia to determine adrenaline, noradrenaline, dopamine, troponin I (TP I) and creatine kinase MB (CK-MB). The animals were then euthanized by administering 50 mg/kg thiopental sodium ip and heart tissues were collected. Heart tissues were divided into two in the coronal plane. Half of the heart tissues were used for biochemical analysis of malondialdehyde (MDA), total glutathione (tGSH), superoxide dismutase (SOD) and catalase (CAT). The other half was evaluated histopathologically.

### 2.5 Biochemical analyses

#### 2.5.1 Preparation of tissue and serum samples for biochemical analysis

Heart tissues were washed with saline and rapidly crushed in a mortar with the addition of liquid nitrogen. The crushed tissues were transferred to a 50 mM (pH = 7.2) buffer mixture prepared with potassium phosphate monobasic and potassium phosphate dibasic. Homogenates were centrifuged (15,000 rpm, 15 min). After centrifugation, the supernatants were carefully separated. Blood samples were transferred to dry biochemistry tubes and centrifuged at 4,000 rpm for 15 min to separate the serum. The supernatant was collected. The heart samples and the separated serum were stored at −80°C until analysis.

#### 2.5.2 Determination of MDA, GSH, SOD, CAT, and protein in heart tissue

MDA tGSH, SOD, and CAT analyses were performed in heart tissues. Determination of MDA (Cat no: 706002), GSH (Cat no: 703002) and SOD (Cat no: 10009055) in tissue samples was performed using “Enzyme-Linked Immunosorbent Assay” (ELISA) commercial rat kits (Cayman Chemical Company, Ann Arbor, MI, United States). Rat CAT ELISA kits purchased from ELK Biotechnology (Hamburg, Germany) were used for CAT (Cat no: ELK-5986) determination. Manufacturer’s instructions for each assay were followed. The protein concentration in the supernatant was determined by the Bradford method and all tissue values are expressed per gram or milligram of protein (Bradford, 1976).

#### 2.5.3 Determination adrenaline, noradrenaline and dopamine in serum

Seum catecholamine levels were determined by an isocratic system using a serum high-performance liquid chromatography (HPLC) pump (Hewlett Packard Agilent 1,100; Hewlett Packard Enterprise, Spring, United States; flow rate: 1 mL/min; injection volume). A reagent kit was used for the HPLC assay of catecholamines in serum (Chromsystems, Munich, Germany).

#### 2.5.4 Determination TP I and CK-MB in serum

Serum TP I levels were measured by enzyme-linked fluorescence assay using the VIDAS TP I Ultra kit. All steps of the assay were performed automatically on the VIDAS instrument using the ready-to-use reagents provided in the kit. CK-MB in serum was determined on a Roche/Hitachi Cobas C701 system. All steps of the assay were performed by immune UV assay using prepared assay reagents according to the procedure.

### 2.6 Histopathological analysis

During sacrification, myocardial tissue samples were collected from each individual. Two transverse specimens were randomly excised near the apex from the left and right heart ventricles. Heart tissue samples were fixed with 10% formaldehyde and washed under tap water. Samples were dehydrated by passing through graded alcohol series. After treatment with xylol, the tissues were embedded in paraffin. Four to five-micron sections were taken from the paraffin blocks and stained with haematoxylin-eosin (H & E). DP2-SAL firmware and a light microscope (Olympus Inc., Tokyo, Japan) were used for analysis. Histopathological analysis was performed on a total of 30 rats, 6 rats in each group. One central and five peripheral areas were selected from serial sections for each rat. Histopathological changes of tissues were scored from 0 to 3 for the presence of degeneration, myofibre disorganization, capillary dilatation/congestion, and interstitial edema (0, normal; 1, mild damage; 2, moderate damage; 3, severe damage. The evaluation was performed by a pathologist blinded to the groups.

### 2.7 Statistical analyses

“SPSS for Windows, 22.0” program was used for the statistical procedures. The quantitative data were expressed as “mean ± standard deviation” (X±SD). The extent to which the experimental intervention differentiated parameter and biomarker values was calculated by Cohen’s d test (Cohen, 1988). Kolmogorov-Smirnov and Levene tests indicated that the data were normally distributed, and variances were homogeneous. Therefore, the analysis used a one-way ANOVA test followed by a *post hoc* Tukey test. The semiquantitative histopathological scoring data were analysed by Kruskal Wallis and *post hoc* Man Whitney U tests. The semiquantitative data were expressed as median (quartile 1- quartile 3) and X±SD. *p* < 0.05 was accepted to be significant.

## 3 Results

### 3.1 Biochemical results

#### 3.1.1 MDA, tGSH, SOD, and CAT levels in heart tissue

**FIGURE 1:**
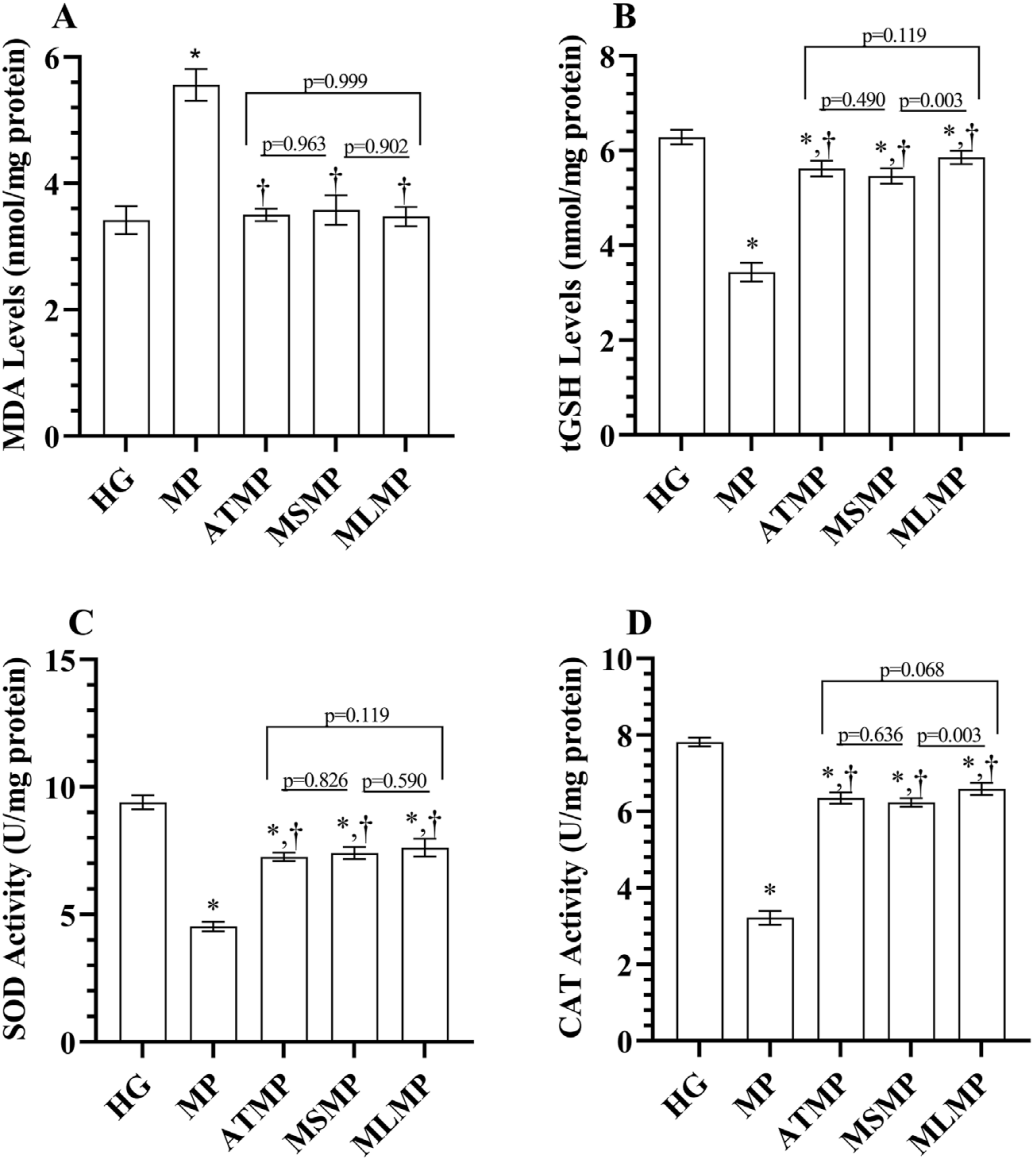
**(A–D)** Analysis of oxidant and antioxidant parameters obtained from heart tissues. Bars indicate mean ± standard deviation, n = 6. *p < 0.001 vs. HG, ^†^ *p* < 0.001 vs. MP, MDA, malondialdehyde; tGSH, total glutathione; SOD, superoxide dismutase; CAT, catalase; HG, healthy; MP, methylphenidate; ATMP, adenosine triphosphate + methylphenidate; MSMP, metyrosine + methylphenidate; MLMP, melatonin + methylphenidate.

As shown in Figure 1A; Table 1, the MDA level was significantly higher in the MP group’s heart tissues than healthy group (*p* < 0.001, Cohen d: 9.068). MDA levels were lower in ATMP, MSMP, and MLMG groups compared to MP group (*p* < 0.001, Cohen’s d: 10.84, 7.92, 9.91, respectively). MDA levels were close to each other in rats given ATP, metyrosine, and melatonin (*p* > 0.05, Cohen’s d: ATMG vs. MSMP; 0.43, ATMP vs. MLMP; 0.19, MSMP vs. MLMP; 0.17). MDA levels in the ATMP, MSMP, and MLMP groups were similar to HG (*p* > 0.05, Cohen’s d: 0.15, 0.68, 0.30, respectively).

**TABLE 1:** Analysis of biochemical data obtained from heart tissues and serum samples.

Biochemical variables	Experimental groups				
	HG	MP	ATMP	MSMP	MLMP
	Mean ± standard deviation				
MDA	3.42 ± 0.22	5.56 ± 0.25*	3.50 ± 0.10	3.58 ± 0.24	3.48 ± 0.15
tGSH	6.29 ± 0.15	3.43 ± 0.20*	5.62 ± 0.16*^,†^	5.47 ± 0.16*^,†^	5.86 ± 1.14*^,†^
SOD	9.40 ± 0.28	4.53 ± 0.18*	7.25 ± 0.16*^,†^	7.41 ± 0.23*^,†^	7.62 ± 0.35*^,†^
CAT	7.82 ± 0.11	3.22 ± 0.18*	6.35 ± 0.15*^,†^	6.24 ± 0.11*^,†^	6.59 ± 0.16*^,†^
Adrenalin	332.67 ± 11.36	567.67 ± 15.72*	559.50 ± 17.74*	328.50 ± 10.41^†^	557.33 ± 12.06*
Noradrenaline	472.67 ± 9.87	777.67 ± 8.19*	771.33 ± 13.82*	476.83 ± 10.98^†^	765.17 ± 12.09*
Dopamine	485.00 ± 19.30	786.67 ± 5.01*	786.67 ± 18.61*	483.17 ± 17.16^†^	781.83 ± 22.00*
TP I	0.025 ± 0.003	0.087 ± 0.006*	0.031 ± 0.003^†^	0.059 ± 0.004*^,†^	0.056 ± 0.005*^,†^
CK-MB	48.50 ± 6.29	88.00 ± 6.16*	50.67 ± 3.50^†^	78.33 ± 5.20*^,†^	78.83 ± 5.67*

*p < 0.05 vs. HG, ^†^: *p* < 0.05 vs. MP. MDA, malondialdehyde; tGSH, total glutathione; SOD, superoxide dismutase; CAT, catalase; TP I, Troponin I; CK-MB, creatine kinase MB; HG, healthy; MP, methylphenidate; ATMP, adenosine triphosphate + methylphenidate; MSMP, metyrosine + methylphenidate; MLMP, melatonin + methylphenidate. Statistical analysis was done with one way ANOVA, followed by a *post hoc* Tukey test. *p* < 0.05 was considered significant.

The tGSH levels in the MP group were lower than in the HG group (*p* < 0.001, Cohen’s d: 15.85). It was observed that tGSH levels in ATMP, MSMP, and MLMG groups were higher than MP group (*p* < 0.001, Cohen’s d: 12.17, 11.30, 14.27, respectively). Although the tGSH levels of the ATMP group were statistically close to MLMP and MSMP, the effect size data showed a large difference (*p* > 0.05, Cohen’s d: 1.49, 0.93, respectively). tGSH levels in the MLMP group were higher than in the MSMP group (*p* = 0.003, Cohen’s d: 2.62) (Figure 1B; Table 1). The SOD activity was decreased in the heart tissues of rats in the MP group compared to HG (*p* < 0.001, Cohen’s d: 20.70). SOD activity was higher in ATMP, MSMP, and MLMG groups compared to MP group (*p* < 0.001, Cohen’s d: 15.99, 13.69, 11.03, respectively). SOD activities were statistically similar in ATMP, MSMP and MLMP groups, but Cohen’s d test showed that there was a difference in the data between the groups (*p* > 0.05, Cohen’s d: ATMG vs. MSMP; 0.77, ATMP vs. MLMP; 1.36, MSMP vs. MLMP; 0.7) (Figure 1C; Table 1).

CAT activities in the MP group were also decreased compared to the healthy group (*p* < 0.001, Cohen’s d: 30.65). CAT activities were found to be higher in rats given ATP, metirozin and melatonin compared to rats in the MP group (*p* < 0.001, Cohen’s d: 15.99, 13.69, 11.03, respectively). Although no difference was detected in the statistical analysis of CAT data obtained from the ATMP group and MSMP and MLMP data (*p* > 0.05), Cohen’s d-test revealed the difference between the groups (0.93, 1.56, respectively). Ayrıca, melatonin verilen ratlardaki CAT aktivitelerinin metirozin verilenlerden daha yüksek olduğu görüldü (*p* = 0.003, Cohen’s d: 2.5) (Figure 1D; Table 1).

#### 3.1.2 Adrenaline, noradrenaline, and dopamine levels in serum

**FIGURE 2:**
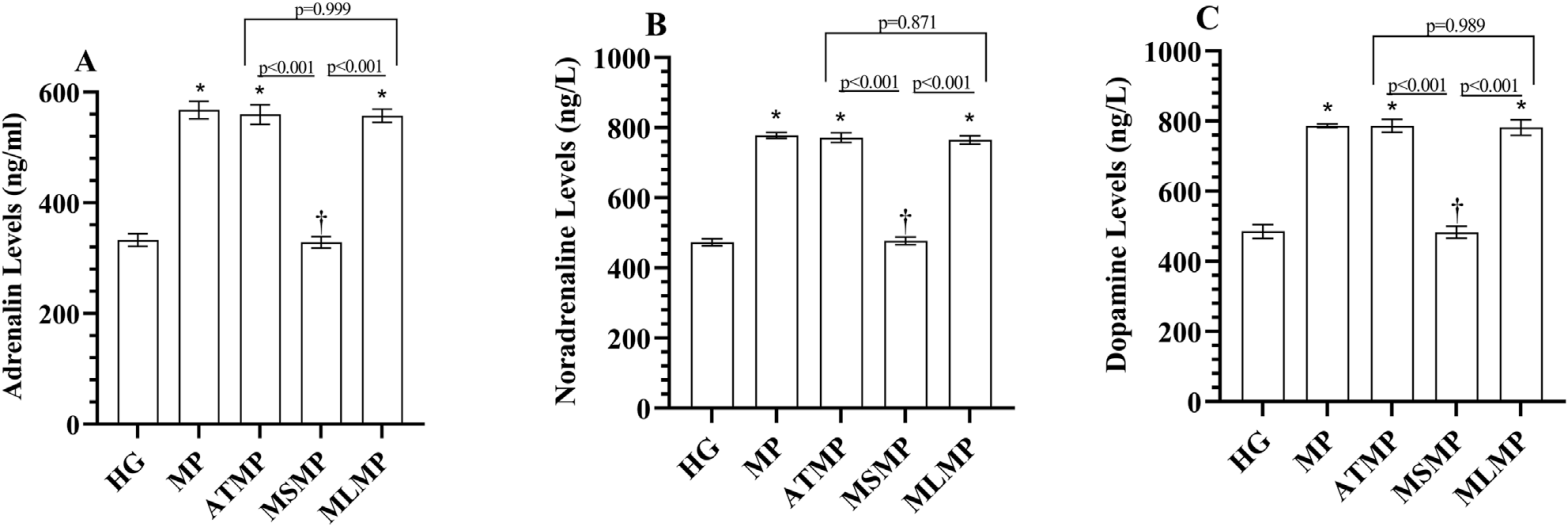
**(A–C)** Analysis of serum catecholamine levels. Bars indicate mean ± standard deviation, n = 6. * *p* < 0.001 vs. HG, ^†^ *p* < 0.001 vs. MP, HG, healthy; MP, methylphenidate; ATMP, adenosine triphosphate + methylphenidate; MSMP, metyrosine + methylphenidate; MLMP, melatonin + methylphenidate.

As shown in Figures 2A–C; Table 1, serum adrenaline, noradrenaline and dopamine levels were higher in the MP group than in the HG group (*p* < 0.001, Cohen’s d: 17.15, 33.62, 21.39, respectively). Adrenaline, noradrenaline, and dopamine levels measured in the MSMP group were lower than in the MP group (*p* < 0.001, Cohen’s d: 18.54, 31.05, 24.01, respectively). The statistical difference between adrenaline, noradrenaline, and dopamine levels in ATMP (Cohen’s d: 0.49, 0.56, 0, respectively) and MLMP (Cohen’s d: 0.74, 1.21, 0.3, respectively) groups and MP group was not significant (*p* > 0.05).

#### 3.1.3 TP I and CK-MB levels in serum

**FIGURE 3:**
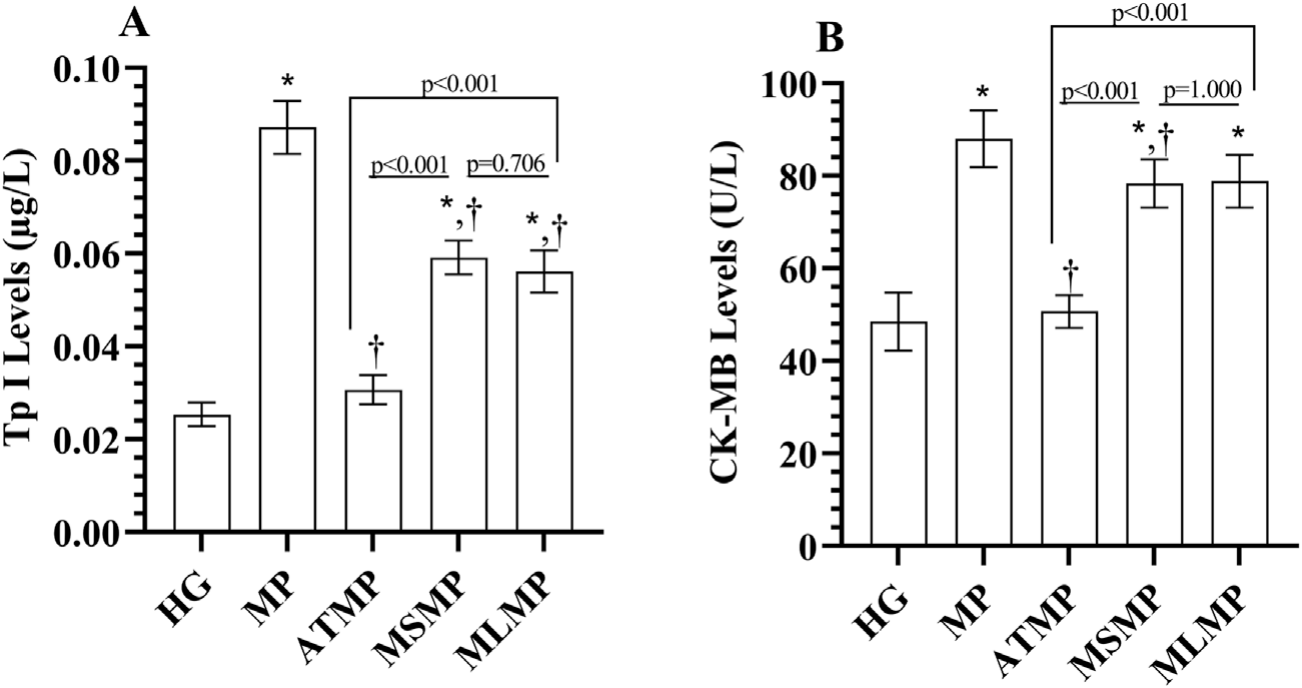
**(A, B)** Analysis of serum TP I and CK-MB levels. Bars indicate mean ± standard deviation, n = 6. * *p* < 0.001 vs. HG, ^†^ *p* < 0.05 vs. MP, TP I, Troponin; CK-MB, creatine kinase MB; HG, healthy; MP, methylphenidate; ATMP, adenosine triphosphate + methylphenidate; MSMP, metyrosine + methylphenidate; MLMP, melatonin + methylphenidate.

Serum TP I levels of the MP group were increased compared to HG (*p* < 0001, Cohen’s d: 13.17). TP I levels were lower in ATMP, MSMP, and MLMP groups compared to MP (*p* < 0.001, Cohen’s d: 12.02, 5.60, 5.64, respectively). In addition, TP I levels were lower in the ATMP group than in the MSMP and MLMP groups (*p* < 0.001, Cohen’s d: 8.14, 6.38, respectively). Although there was a difference in effect size for TP I levels between MSMP and MLMP, the statistical difference was not significant (*p* = 0.705, Cohen’s d: 0.67) (Figure 3A; Table 1).

Similarly, serum CK-MB activity in the MP group was increased compared to HG (*p* < 0.001, 6.34). CK-MB activity in ATMP (*p* < 0.001, Cohen’s d: 7.47) and MSMP (*p* = 0.038, Cohen’s d: 1.70) groups was lower than MP. However, CK-MB values of MSMP and MP groups were similar (*p* = 0.053, Cohen’s d: 1.55). CK-MB levels in ATMP group were lower than those in MSMP (*p* < 0.001, Cohen’s d: 6.24) and MLMP (*p* < 0.001, Cohen’s d: 5.99) groups. CK-MB levels were similar in methyrosine and melatonin-treated rats (*p* = 1.000, Cohen’s d: 0.09) (Figure 3B; Table 1).

### 3.2 Histopathologic results

**FIGURE 4:**
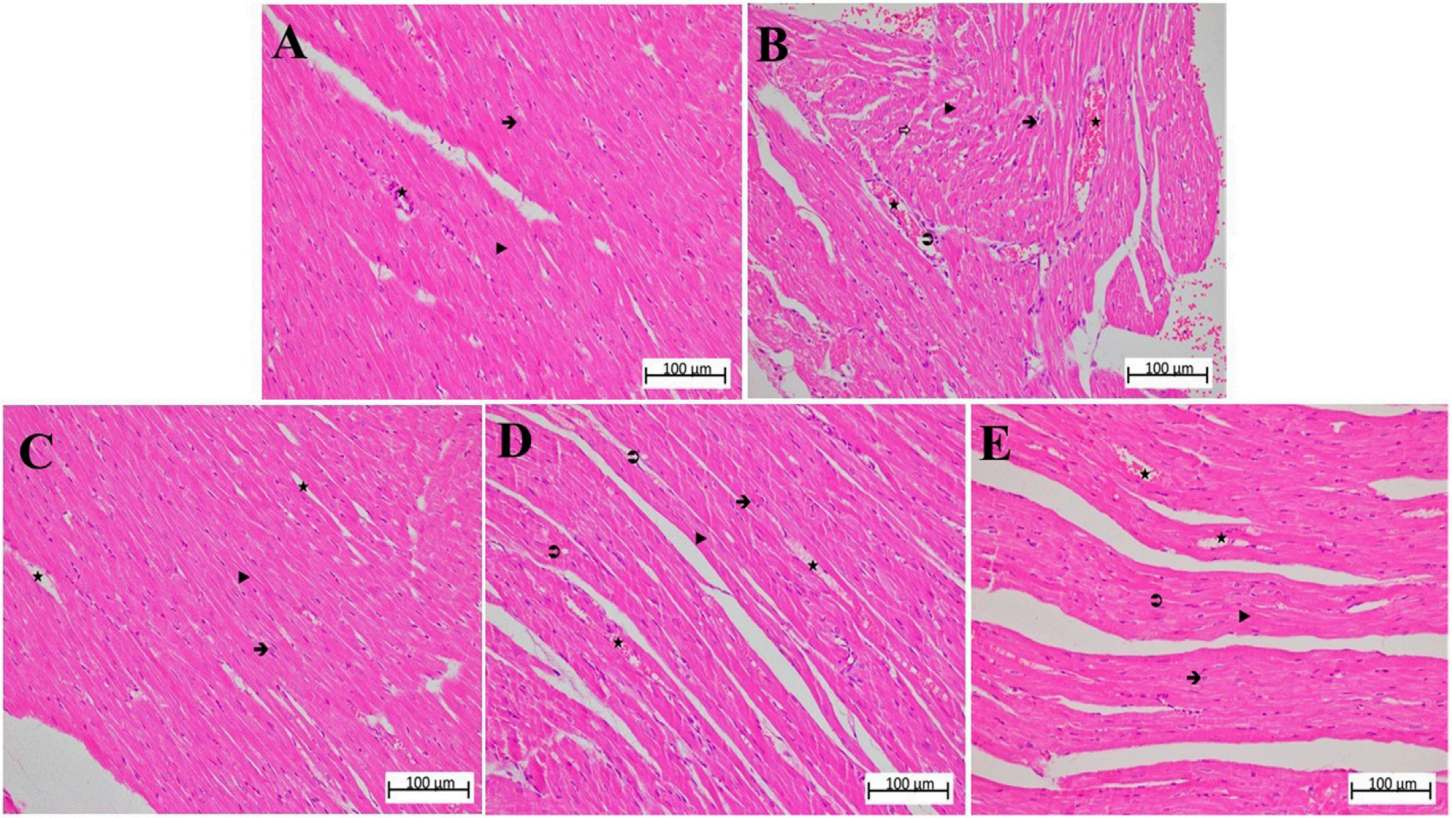
**(A–E)** Hematoxylin-eosin stained sections of heart tissue in different animal groups. The magnification of **(A–E)** is ×200. **(A)** control group heart sections showing normal appearance of cardiac myofibers, interstitial area, and blood vessels. **(B)** MP group sections showing degeneration of muscle fibers, diffuse inflammatory cell infiltration, grade 3 interstitial edema, and congestion. **(C)** ATMP group sections were close to normal architecture of myocytes, grade 2 congestion in blood vessels, and reduction of edema. **(D)** sporadical eosinophilic degeneration and disruption in myocytes and grade 2 congestion in blood vessels in MSMP group. **(E)** MLMP group, generally normal cardiomyocytes and moderately congested blood vessels were seen. 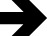: cardiomyocyte nucleus, 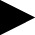: myofibers, 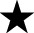: blood vessel, 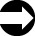: interstitial edema, 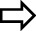: polimorphonuclear cell infiltration, HG, healthy; MP, methylphenidate; ATMP, adenosine triphosphate + methylphenidate; MSMP, metyrosine + methylphenidate; MLMP, melatonin + methylphenidate.

According to the microscopic examination of cardiac muscle tissue, the cardiomyocyte nuclei in the HG control group were single, oval, prominent, and centrally positioned. The transverse lines of the cardiomyocytes were visible, and the myofibrils were interconnected with parallel branches and well organized. Likewise, the connective tissue structure surrounding the cardiomyocytes and blood capillaries was normal (Figure 4A; Table 2).

**TABLE 2:** Analysis of histopathological data obtained from heart tissues.

Histopathological variables	Experimental groups				
	HG	MP	ATMP	MSMP	MLMP
	Median (quartile 1- quartile 3) Mean ± standard deviation				
Degeneration	0 (0–0) (0 ± 0)	3 (3–3)* 2.86 ± 0.35	1 (0–1)*^,†^ 0.78 ± 0.54	2 (1–2)*^,†,‡^ 1.67 ± 0.48	1 (1–2)*^,†^ 1.42 ± 0.55
Myofiber irregularity	0 (0–0) (0 ± 0)	3 (3–3)* 2.78 ± 0.42	1 (0–1)^†^ 0.72 ± 0.62	2 (2–3)*^,†,‡^ 2.06 ± 0.72	2 (2–2)*^,†,‡^ 1.97 ± 0.45
Dilatation/Congestion	0 (0–0) (0 ± 0)	3 (2.25–3)* 2.75 ± 0.44	2 (1–2)*^,†^ 1.44 ± 0.77	2 (1–2)*^,†^ 1.81 ± 0.58	2 (1–2)*^,†^ 1.58 ± 0.81
Insterstitial edema	0 (0–0) (0 ± 0)	3 (2–3)* 2.56 ± 0.50	1 (0–1)*^,†^ 0.61 ± 0.60	2 (1–2)*^,†,‡^ 1.72 ± 0.45	1 (1–1)*^,†^ 0.92 ± 0.60

Histopathological grading: 0; no damage, 1, mild damage, 2; moderate damage, 3; severe damage. * *p* < 0.05 vs. HG, ^†^ *p* < 0.05 vs. MP, ^‡^ *p* < 0.05 vs. ATMP, HG, healthy; MP, methylphenidate; ATMP, adenosine triphosphate + methylphenidate; MSMP, metyrosine + methylphenidate; MLMP, melatonin + methylphenidate Statistical analysis was performed with Kruskal Wallis test followed by a *post hoc* Tukey test.

In the MP group, in which the severity of damage was determined as Grade-3, it was determined that the cardiomyocyte nuclei lost their normal shapes. It was observed that the nuclei became smaller, some of them were stained darker than normal and some of them remained pale. In some myofibrils, there were evidence of eosinophilia, degeneration, and interstitial edema. There was a disruption in both the arrangement and general structure of myofibrils. As a result, the connecting units of the cells lost their distinctness, and the collateral branches exhibited a wavy shape. There was a significant amount of dilation and congestion of blood capillaries, as well as polymorphonuclear cells in the perivascular area (Figure 4B; Table 2).

The nuclei of cardiomyocytes and the shape and organization of myofibrils of the ATMP group were generally normal (grade 0–1). There was a moderate dilated capillary pattern and mild congestion of the blood vessels (grade 2), but there was a substantial reduction in edema within the interstitial tissues (grade 0–1). No significant polymorphonuclear cell infiltration was observed (Figure 4C; Table 2).

MSMP group, the cell nuclei of cardiomyocytes were generally normal in size and shape, although some still showed eosinophilic degeneration (grade 2). In some myofibrils, disruption of shape and organization persisted (grade 3). Blood capillaries were moderately dilated and congested (grade 2), while interstitial edema was mild to moderate (grade 1–2) (Figure 4D; Table 2).

In the MLMP group, cardiomyocytes were observed to show eosinophilic degeneration (grade 1–2). While some of the myofibrils were still deformed and disorganized (grade 2), blood capillaries were mildly to moderately dilated and congested (grade 1–2) and tissue-wide edema was mild (grade 1) (Figure 4E; Table 2).

## 4 Discussion

In the present study, it has been investigated whether methylphenidate administration is cardiotoxic in rats. The potential protective properties of ATP, metyrosine, and melatonin against the possible cardiotoxicity of methylphenidate were evaluated. For this purpose, oxidant/antioxidant, catecholamine, and cardiac damage marker levels were determined. In addition, heart tissues were evaluated histopathologically. In the methylphenidate group, an increase in oxidants and catecholamines and a decrease in antioxidants were observed in heart tissues compared to the control group. In addition, an increase in markers of cardiac damage was also detected. Histopathological analysis revealed severe tissue damage in the heart tissues of the methylphenidate group. ATP, metyrosine and melatonin administrations were effective in preventing methylphenidate-induced biochemical and histopathological changes. Howewer, only metyrosine was able to inhibit catecholamine increase. In addition, several parameters evaluated in ATP-treated rats were found to be closer to normal compared to metyrosine and melatonin-treated rats.

There have been reports in the literature regarding oxidative stress induced by methylphenidate in various tissues, including cardiac tissue, however, the underlying mechanisms remain unclear (Guney et al., 2015; Loureiro-Vieira et al., 2018; Martins et al., 2006; Motaghinejad et al., 2016). In a study carried out on young rats, it was reported that chronic use of methylphenidate caused oxidative damage in brain tissue (Martins et al., 2006). In another study, chronic use was found to cause oxidative stress and inflammation in the hippocampus (Motaghinejad et al., 2016). In addition, Guney et al. (2015) found that methylphenidate decreased oxidant levels and increased antioxidant levels in children and adolescents. In this study, the levels of endogenous oxidant/antioxidant parameters such as MDA, tGSH, SOD and CAT were measured to determine the oxidative stress induced by methylphenidate in cardiac tissue. In the heart tissue of methylphenidate-applied animals, MDA levels were higher and tGSH, SOD and CAT levels were lower according to the HG. MDA has been known as the end product of peroxidation of polyunsaturated fatty acids (LPO) in cells and an increase in ROS production causes excessive production of MDA (Lu et al., 2024; Yıldırım et al., 2023). On the other hand, enzymatic and nonenzymatic antioxidants such as GSH, SOD and CAT neutralize ROS and their toxic products and render them harmless (Lu et al., 2024; Motaghinejad et al., 2016; Yıldırım et al., 2023). These data suggest that methylphenidate induces a significant oxidative stress in cardiac tissue. Methylphenidate exerts its pharmacological effects both by blocking norepinephrine and dopamine reuptake in presynaptic neurons and by stimulating 5-HT1A receptors (Bieś et al., 2023; Markowitz et al., 2009). Increased levels of dopamine and norepinephrine in the prefrontal cortex, which controls attention and cognitive functions, are involved in the therapeutic effect (Quintero et al., 2022). In this study, it was observed that the amount of adrenaline, noradrenaline and dopamine in the blood serum of animals in the methylphenidate group increased. Numerous studies have suggested that elevated catecholamine levels in tissues are positively correlated with markers of oxidative stress and negatively correlated with markers of antioxidant protection (Sah et al., 2024; Turková at al., 2013). On the other hand, it has been reported in the literature that methylphenidate may trigger cardiomyopathy directly via alpha and beta receptors (Dadfarmay and Dixon, 2009). Serum TP I and CK-MB levels, which we used to evaluate cardiac damage in the study, were found to be increased in the methylphenidate group. As known, TP I is a heart-specific regulatory protein that is an integral part of contraction in the heart muscle (Mohebi et al., 2022). It is widely used as a diagnostic and prognostic indicator in the management of myocarditis, myocardial infarction, and acute coronary syndrome (Tveit et al., 2022). Methylphenidate has previously been reported to cause an increase in cardiac troponin (Bieś et al., 2023). On the other hand, creatine kinase is an enzyme that catalyzes the conversion of creatinine and ATP to creatine phosphate and ADP (Aydin at al., 2019). Measurement of CK-MB activity is considered a good indicator of cardiac muscle damage and has been used to diagnose acute myocardial infarction for years (Zhang at al., 2024). Methylphenidate has increased creatine kinase activity in humans and experimental animals (Nagaoka et al., 2024). In this study, the morphology of cardiomyocytes was disrupted after the administration of methylphenidate. The arrangement and general structure of cardiomyocyte myofibrils were found to be disrupted, and the capillaries were highly dilated and congested. According to the histopathologic findings from the methylphenidate group, the cardio-pathologic morphologies were similar to those previously reported (Brayson et al., 2020; Kuprytė et al., 2023; Lushnikova et al., 2004).

In our study, the protective effects of ATP against the cardiotoxic effect of methylphenidate were investigated. ATP is known for its role in intracellular energy metabolism. However, it is an important extracellular signaling molecule. Outside the cell, ATP regulates many cellular functions through purinergic P2 receptors (Patel et al., 2018). The literature is dominated by the idea that ATP cannot cross the cell membrane (Chaudry, 1982). However, systemic and oral administration has been reported to expand ATP pools. In addition, infused ATP has been shown to act through purinergic receptors (Jäger et al., 2014). Methylphenidate has previously been reported to decrease cells ATP levels (Foschiera et al., 2022). In a study including the effect of methylphenidate on the heart, heart damage was detected. However, there was no significant decrease in ATP levels in heart tissues (Loureiro-Vieira et al., 2018). It has been shown that chronic methylphenidate administration causes a decrease in hippocampal ATP levels and ATP synthase activity in brain tissue with high ATP consumption such as the heart. The authors also pointed out that impairments in ATP production may result in tissue damage (Liu et al., 2016; Schmitz et al., 2017). The decrease in ATP levels negatively affects the synthesis of cellular antioxidants and the neutralization of ROS (Saquet et al., 2000; Yi et al., 2010). On the other hand, intracellular ATP depletion has been reported to cause an increase in ROS production (Agalakova and Gusev, 2012). Our results revealed that the redox balance, which was disturbed in favor of methylphenidate-induced oxidants, was changed in favor of antioxidants by exogenous ATP administration. Cardiomyocytes require high levels of ATP (Sudi et al., 2023). Therefore, a stable concentration of ATP molecule is an important component of the frequency and force of cardiac contraction (Long, Yang and Yang, 2015). The study by Beard et al. (2022) reported that a decrease in ATP level decreased the force of cardiac contraction and impaired pumping function. Another study revealed that oxidative damage and associated ATP depletion contributed to the development of left ventricular dysfunction in cardiomyopathy (Ichihara et al., 2017). In this study, ATP was observed to increase these levels closer to the values of the healthy control group. A previous study by our team demonstrated the cardioprotective effects of ATP in rats exposed to bevacizumab-induced cardiotoxicity (Yıldırım et al., 2023). In addition, a study by Lian et al. (2016) also emphasized that ATP exerted cardioprotective effects against myocardial ischemia-reperfusion injury. Markers of cardiac damage were lower in rats receiving ATP than in rats in the methylphenidate, metyrosine and melatonin groups. Our histopathological findings also confirmed the cardioprotective effects of ATP.

Furthermore, we investigated the protective effects of metyrosine against methylphenidate cardiotoxicity in light of information that suggests that methylphenidate cardiotoxicity may result from increased sympathetic activity (Lamberti et al., 2015). In the study, it was determined that metirosine exhibited antioxidant activity by preventing the increase of methylphenidate-induced oxidants (MDA) and antioxidant depletion (GSH, SOD, CAT). Banerjee et al. (2003) also emphasized the importance of oxidative stress in catecholamine-induced myocardial necrosis. They argued that catecholamines undergo rapid oxidation and oxidative products are responsible for myocardial changes. In a study that confirmed our findings, it was reported that metyrosine exhibited antioxidant activity, and protected cardiac tissue against oxidative stress (Ahiskalioglu et al., 2015). The improvement in biochemical and histopathological data with metyrosine administration confirms the role of catecholamine increase and related oxidative damage in methylphenidate cardiotoxicity. In addition, serum adrenaline, noradrenaline, and dopamine levels in the methylphenidate group were lower than those in the methylphenidate group. Catecholamine levels in the ATP and melatonin groups were similar to the methylphenidate group. However, there are contradictions in the association of metyrosine, a catecholamine synthesis inhibitor, with catecholamine levels (Elliott et al., 2024). However, methylphenidate-induced TP I and CK-MB elevations were significantly inhibited by metyrosine, although not as much as ATP. Metyrosine, however, has been found to protect cardiac tissue against ketamine-induced oxidative stress and inhibit TP I and CK-MB elevations (Ahiskalioglu et al., 2015). The severity of histological damage in the metyrosine group was moderate, and the damage was relatively greater than in the ATP and melatonin groups. Although metyrosine inhibited catecholamine increase and showed similar antioxidant activity to ATP, it was not as successful as ATP, especially on markers of cardiac damage. These findings contradict the information on the effect of catecholamine elevation on methylphenidate cardiotoxicity (Torres-Acosta et al., 2020). However, it may mean that ATP exerts a protective effect on methylphenidate cardiotoxicity through other pathways that were not included in our study. On the other hand, considering that the mechanism of action of methylphenidate is especially through catecholamine increase, the use of metyrosine as a protective agent may also reduce the efficacy of methylphenidate.

The corrective effect of melatonin on oxidative and antioxidative markers in cardiac tissue was relatively better than that of ATP and metyrosine, although the difference was not statistically significant. It has been reported in the literature that melatonin has a strong ROS scavenging capacity and also induces the expression of antioxidant genes including glutathione peroxidase, SOD and CAT (Zhang et al., 2023). Furthermore, the solubility of melatonin in both water and lipids allows it to easily pass through the cell membrane. This property enables it to enter heart cells and protect cellular macromolecules from oxidative attacks (Ahmed et al., 2005). *In vitro* and *in vivo* studies show that melatonin increases the expression of antioxidant agents such as SOD and tGSH and inhibits cardiotoxic effects by decreasing MDA levels (Abouelella et al., 2024; Durdagi et al., 2021; Zhang et al., 2023). In our study, it was observed that melatonin could not prevent the methylphenidate-induced increase in serum catecholamines. Our findings contradict the information of Komatsabura et al. that melatonin prevents catecholamine synthesis by suppressing the production of catecholamine synthases and inhibiting voltage-sensitive calcium channels (Komatsubara et al., 2017). Melatonin has been previously used against doxorubicin oxidative damage and has been reported to suppress the increase in TPI and to be cardioprotective (Ahmed et al., 2005). In addition, histopathological evaluation of the melatonin group showed that the severity of cardiac damage was mild to moderate. Although it showed similar effects to ATP in terms of antioxidative activity, cardiac damage parameters, and histopathological damage were higher than in the ATP group. This again supports our idea that ATP has a cardioprotective effect through different mechanisms.

### 4.1 Limitations

The limitations of the study are that electrocardiography was not performed to evaluate chronotropic function, and an invitro isolated heart study or echocardiography was not performed to evaluate ionotropic function. In addition, the fact that tissue ATP levels were not studied is among the limitations of our study.

## 5 Conclusion

Methylphenidate induced cardiotoxic effects in rats, including an abnormal oxidant/antioxidant milieu, abnormal levels of cardiac markers, and pathological changes to the heart tissue. ATP, metyrosine, and melatonin showed approximately similar levels of antioxidant activity against methylphenidate-induced oxidative stress. Cardiac damage parameters were lower in the ATP group than in the methylphenidate, metirozine, and melatonin groups. ATP and melatonin were more effective than metirozine in preventing methylphenidate-induced histopathological damage. Catecholamine increase, which we attributed to methylphenidate use, was decreased in the metirozine group. However, this finding did not place metyrosine ahead of ATP and melatonin in terms of cardioprotective effect. We hope our study will lead to clinical studies testing ATP and melatonin in protection against methylphenidate-induced cardiotoxicity.

## Data Availability

The raw data supporting the conclusions of this article will be made available by the authors, without undue reservation.
